# Prevalence of specific anti-skin autoantibodies in a cohort of patients with inherited epidermolysis bullosa

**DOI:** 10.1186/1750-1172-8-132

**Published:** 2013-09-04

**Authors:** Marilina Tampoia, Domenico Bonamonte, Angela Filoni, Lucrezia Garofalo, Maria Grazia Morgese, Luigia Brunetti, Chiara Di Giorgio, Giuseppina Annicchiarico

**Affiliations:** 1 Laboratory of Clinical Pathology, University Hospital of Bari, Piazza Giulio Cesare 11, Bari 70124, Italy; 2 Department of Biomedical Sciences and Human Oncology, Dermatology Department, University Hospital of Bari, Bari, Italy; 3 Department of Clinical and Experimental Medicine, University of Foggia, Foggia, Italy; 4 Pediatric Department “B. Trambusti”, University Hospital of Bari, Bari, Italy; 5 Department of Emergency and Organ Transplantation, University of Bari, Bari, Italy; 6 Regional Coordination for Rare Diseases-Ares Puglia, Bari, Italy

**Keywords:** Inherited epidermolysis bullosa, Dystrophic epidermolysis bullosa, Anti-skin autoantibodies, Type VII collagen, BP180, BP230, ELISA, Birmingham Epidermolysis Bullosa Severity score

## Abstract

**Background:**

Inherited epidermolysis bullosa (EB) is a group of skin diseases characterized by blistering of the skin and mucous membranes.

There are four major types of EB (EB simplex, junctional EB, dystrophic EB and Kindler syndrome) caused by different gene mutations. Dystrophic EB is derived from mutations in the type VII collagen gene (COL7A1), encoding a protein which is the predominant component of the anchoring fibrils at the dermal-epidermal junction.

For the first time in literature, we have evaluated the presence of anti-skin autoantibodies in a wider cohort of patients suffering from inherited EB and ascertained whether they may be a marker of disease activity.

**Methods:**

Sera from patients with inherited EB, 17 with recessive dystrophic EB (RDEB), 10 with EB simplex (EBS) were analysed. As much as 20 patients with pemphigus vulgaris, 21 patients with bullous pemphigoid and 20 healthy subjects were used as controls.

Anti-skin autoantibodies were tested in all samples with the Indirect Immunofluorescence (IIF) method and the currently available ELISA method in order to detect anti-type VII collagen, anti-BP180 and anti-BP230 autoantibodies.

**Results:**

The mean concentrations of anti-type VII collagen autoantibodies titres, anti-BP180 and anti-BP230 autoantibodies were statistically higher in RDEB patients than in EBS patients.

The sensitivity and specificity of the anti-type VII collagen ELISA test were 88.2% and 96.7%. The Birmingham Epidermolysis Bullosa Severity score, which is used to evaluate the severity of the disease, correlated with anti-skin autoantibodies titres.

**Conclusions:**

The precise pathogenic role of circulating anti-skin autoantibodies in RDEB is unclear. There is a higher prevalence of both anti-type VII collagen and other autoantibodies in patients with RDEB, but their presence can be interpreted as an epiphenomenon.

## Background

Epidermolysis bullosa (EB) is a group of both acquired and inherited diseases characterized by blistering of the skin and mucous membranes, in response to little or no apparent trauma [1].

In inherited EB, the phenotypic manifestations of this disease can be very different; in the milder forms, there is a lifelong blistering tendency with no impact on the overall longevity of the affected subject, while in the most severe forms children die during the early postnatal period from metabolic perturbations, dehydration, and sepsis [2].

Inherited EB has typically been classified into four main subtypes; these differ not only phenotypically and genotypically but, more importantly, by the site of ultrastructural distruption or cleavage. Tissue separation occurs within the intraepidermal keratinocyte cytoplasm in EB simplex (EBS), through the lamina lucida in junctional EB (JEB) or in the sublamina densa in dystrophic EB (DEB) [3]; in Kindler syndrome, multiple cleavage planes may be seen within the same sample of skin [4].

Inherited EB is now known to result from more than 1100 mutations, encompassing more than 14 distinct structural genes expressed within the cutaneous basement membrane zone (BMZ) at the dermoepidermal junction [5,6]. After the initial clinical evaluation, based on careful examination of cutaneous and extracutaneous manifestations and inheritance pattern, the diagnosis is confirmed by the use of three main techniques: immunofluorescence mapping (IFM), transmission electron microscopy (TEM), which can both be used to identify the level of skin cleavage, and mutation analysis [7]. TEM is considered the gold standard laboratory test for differentiation between the various forms of EB. The primary advantage is that it can visualize ultra-structural abnormalities and may be particularly useful in patients with mild DEB or EBS. However, TEM is time-consuming and expensive, and the results are, to a high degree, operator-dependent and sometimes inaccurate [8]. The diagnostic precision of IFM is similar to that of TEM, with the advantage that it is simpler and faster both to perform and to interpret. A previous study has shown that IFM is more sensitive (97% vs. 71%) and specific (100% vs. 81%) than TEM [9].

Mutation analysis is an excellent technique to confirm the diagnosis for the various subtypes of EB as well as to provide information that might be useful for prenatal diagnosis and someday for genetic therapy [10].

### Dystrophic EB and type VII collagen

Dystrophic EB (DEB) forms, also known as dermolytic forms, are characterized by tense blisters and erosions that heal with extensive, either atrophic or hypertrophic, scarring. The scarring tendency reflects the fact that blister formation occurs below the level of the lamina densa of the BMZ of the papillary derma. The spectrum of clinical severity in DEB is, however, highly variable, with milder forms of the disease showing a limited tendency to blistering or nail involvement and severe forms where the gastrointestinal tract, particularly the oesophagus, is affected by blistering and scarring. All forms result from mutations within type VII collagen gene, COL7A1 [10].

The type VII collagen, the main constituent of the anchoring fibrils, is composed of a triple-helical domain flanked by a large 145 kDa non-collagenous amino-terminal (NC-1) domain and a small 34 kDa non-collagenous carboxyl-terminal (NC-2) domain [11,12]. In patients with DEB, ultrastructural analysis of skin demonstrates the presence of altered anchoring fibrils, reduced in number or completely absent in the different forms [13].

### Autoantibodies against type VII collagen

In addition to inherited forms of EB, there is an acquired form of epidermolysis bullosa (EBA), which is caused by an auto-reactive response against collagen VII. Patients with EBA have both tissue-bound and circulating IgG autoantibodies against type VII collagen [14,15]. Specifically, circulating autoantibodies recognize epitopes within the amino-terminal NC-1 domain of type VII collagen [16,17]; only a small number of EBA patients have been described with autoantibodies reactivity against the NC-2 domain [18]. The routine screening tests for the diagnosis of EBA are: direct and indirect immunofluorescence microscopy; in particular, the salt-split skin method. Patients with EBA have immune deposits on the dermal side of salt-split skin, whereas in bullous pemphigoid (BP) deposits are on the epidermal side. However, this method does not distinguish EBA from anti-laminin 332 mucous membrane pemphigoid (MMP) or anti-laminin c1 pemphigoid as these diseases also show immune deposition on the dermal side of salt-split skin. EBA sera recognize the 290 kDa type VII collagen protein in immunoblotting studies; in fact, this reactivity confirms the diagnosis of EBA. However, immunoblotting studies are difficult to carry out because they are time-consuming and technically demanding. Advances in the identification of target antigens and the subsequent development of an increasing number of sensitive and specific assays for the detection of circulating autoantibodies, including recombinant forms of the target antigens and ELISA, allow serological diagnosis in patients with autoimmune skin disorders. Several ELISA systems using recombinant fragments of BP180, BP230, desmoglein1, desmoglein3, have become commercially available and are highly valuable diagnostic tools [19]. ELISAs were also developed to detect autoantibodies in EBA using different recombinant proteins of the NC-1 domain of type VII collagen [20,21]. Previous reports demonstrate that these tests showed high sensitivity and specificity, and in some clinical studies serial titres of anti-type collagen VII from each patient with EBA reflected the disease activity [22-24]. Experimental studies provided evidence that autoantibodies against type VII collagen are pathogenic. When rabbit and human antibodies were passively transferred into mice, they induced subepidermal blister formation and nail dystrophy [25,26]. The active mouse model of EBA was also established by immunizing mice with the recombinant NC-1 domain of murine type VII collagen [27].

Although DEB has been shown to result from mutations in COL7A1, relatively little is known about the mechanistic consequences of the mutations in relation to blistering. To facilitate our understanding of DEB, we have evaluated the presence of anti-skin autoantibodies in a wider cohort of patients suffering from DEB and ascertained whether they correlated with the disease severity.

## Methods

### Patients

Serum samples were obtained from 27 patients with inherited forms of EB, seen at the Dermatological Department of the University Hospital in Bari between January 2010 and December 2012. All patients examined had typical clinical features of EB with a variable phenotype as well as immunofluorescence mapping and transmission electron microscopy findings consistent with EB.

As many as 17 patients had recessive dystrophic EB (RDEB), mean age 21.2 years, range 2-50, 4 male and 13 female; 10 patients had EB simplex (EBS), mean age 17 years, range 1-56, 3 male and 7 female. In all patients, disease severity was evaluated through the Birmingham Epidermolysis Bullosa Severity score (BEBS), which includes 11 items: area of damaged skin, involvement of nails, mouth, eyes, larynx and oesophagus, scarring of hands, skin cancer, chronic wounds, alopecia and nutritional compromise. Area was allocated 50 points, and the 10 other items 5 points each, giving a maximum score of 100 [28]. The BEBS score was performed by a dermatology specialist at blood sample collection.

Diseases control sera were obtained from 41 patients, including 20 patients with pemphigus vulgaris (PV) (mean age 59 years, range 37-85, 6 males, 14 females) and from 21 patients with bullous pemphigoid (BP) (mean age 74 years, range 61-90, 9 males, 12 females).

Control sera were obtained from 20 healthy subjects (mean age 47 years, range 36-65, 6 males and 14 females). All sera were stored at -20°C until assayed.

Patients and control subjects gave written consent to participate in this study, and the Ethics Committee of the hospital approved this study.

### Methods

The detection of circulating anti-skin autoantibodies was performed in all samples with an Indirect Immunofluorescence (IIF) method (Euroimmun, Lubeck, Germany) and with the currently commercially available ELISA methods (MBL, Nagoya, Japan). Anti-type VII collagen ELISA kits were provided free of charge by the manufacturer. All assays were performed at the Laboratory of Clinical Pathology in the University Hospital of Bari.

### IIF method

Slides were made of frozen primate oesophagus section. IIF assays were performed at a serum dilution of 1:10 in phosphate buffered saline (pH 7.3).

Two different fluorescence patterns can be observed on primate esophagus sections: a) anti-intercellular substance (ICS) and anti-basal membrane zone (BMZ). ICS antibodies, which are specific for pemphigus, are directed against prickle cell desmosomes and react with surface antigens of keratinocytes; on tissue sections they display a granular fluorescence of the intercellular matter in the whole stratum spinosum. BMZ autoantibodies, which are specific for pemphigoid diseases or epidermolysis bullosa acquisita, are directed against epidermal basal membrane and generate a fine linear colouring between the stratum basale and the connective tissue.

Samples were scored positive if a fluorescence reaction was observed at 1:10 sample dilution.

### ELISA methods

Autoantibodies detection was performed in all samples with the ELISA systems, each one employing different antigenic proteins coated on the wells of microplates. Assays were run according to the manufacturers’ instructions.

Patients and control sera, diluted 1:100, were added to each well of a microwell plate. For detection of anti-type VII collagen autoantibodies the microplates were coated with human recombinant NC-1 and NC-2 proteins; for detection of anti-BP180 and anti-BP230 autoantibodies, the microplates were coated with recombinant purified BP180-NC16A antigen and recombinant purified BP230-N and BP230-C antigens, respectively. Sera were incubated for 1 hour at RT. After washing to remove any unbound serum proteins, horseradish peroxidise-conjugated anti-human IgG was added and incubated for 1 hour at RT. After another washing step, the peroxidise substrate was added and incubated for 30 min at RT. The acid solution was then added to each well to terminate the enzyme reaction and stabilize the colour development. The value in each sample was obtained by comparing the optical density (OD) of the sample with the OD of the calibrator.

### Statistical analysis

To assess the relationship between clinical features and autoantibodies titres, we divided inherited EB patients into EB simplex (EBS) patients and EB recessive dystrophic (RDEB) patients.

The Kruskal Wallis test was used to compare the titres of each autoantibody assayed by the ELISA methods in patients and in the control group.

A P-value below 0.05 was considered statistically significant. The Unpaired *T* test was used to compare the mean autoantibodies titres between EBS patients and RDEB patients.

The diagnostic sensitivity of ELISA for anti-type VII collagen autoantibodies was calculated in 17 patients with RDEB, and the specificity was calculated in 10 patients with EBS, in 41 control patients with other diseases and in 20 healthy subjects. The cut-off value for positivity was validated and optimised by Receiver Operating Characteristics (ROC).

Correlation analyses were performed by using the Pearson test.

MedCalc software (Mariakerke, Belgium) was used for statistical and ROC curve analysis.

## Results

### Analytical measurements

**Figure 1:**
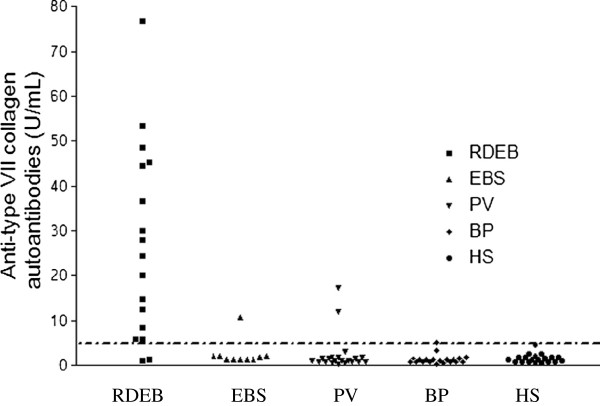
**Distribution of anti-type VII collagen autoantibodies titres.** Distribution of anti-type VII collagen autoantibodies titres, expressed in Units/mL in patients with recessive dystrophic epidermolysis bullosa (RDEB), epidermolysis bullosa simplex (EBS), pemphigus vulgaris (PV), bullous pemphigoid (BP) and healthy subjects (HS). Kruskal Wallis test, P < 0.0001.

In patients with EB, the IIF method did not show an anti-basal membrane zone (BMZ) pattern, as expected. In the present study, all samples were tested at 1:10, in accordance to the recommendations for qualitative IIF given by the manufacturer. However, as the use of only a single dilution may lead to blocking or masking effects in high-titered sera, thus causing false-negative results, sera were retested at 1:100 dilution. At this dilution, no prozone effects were noted, confirming that those sera were true negatives. Detection of antibodies by ELISA test allowed quantitative measurements of autoantibodies serum titres. Figure 1 shows that the mean concentration of anti-type VII collagen autoantibodies titres was statistically higher in patients with RDEB than in patients with EBS, in controls with other diseases and in healthy subjects (test Kruskal Wallis, P < 0.0001). Clinical and serological findings of patients with two different inherited types of EB are reported in Table 1.

**Table 1:** Clinical and serological findings in patients with recessive dystrophic epidermolysis bullosa (RDEB) and with epidermolysis bullosa simplex (EBS)

	**Patients with RDEB**	**Patients with EBS**	
Patients, no.	17	10	
Mean Age	21	17	
Range years	2-50	1-56	
Males/Females	4/13	3/7	
**Birmingham severity score**			
Birmingham severity score, mean	55	5.4	
Birmingham severity score, range	5-90	2-36	
**Autoantibodies titres, mean concentrations**			P*
-Anti-type VII collagen (U/mL)	27.8	2.5	0.0014
-Anti-BP180 (U/mL)	59.4	8.3	0.0028
-Anti-BP230 (U/mL)	51.2	6.6	0.0023

* Unpaired *T* Test, P-value < 0.05 was considered statistically significant.

The mean concentrations of anti-type VII collagen autoantibodies titres were statistically higher in patients with RDEB than in patients with EBS (P =0.0014). The mean concentrations of anti-BP180 and anti-BP230 autoantibodies were also statistically higher in patients with RDEB than in patients with EBS (P = 0.0028 and P = 0.0023, respectively).

**Figure 2:**
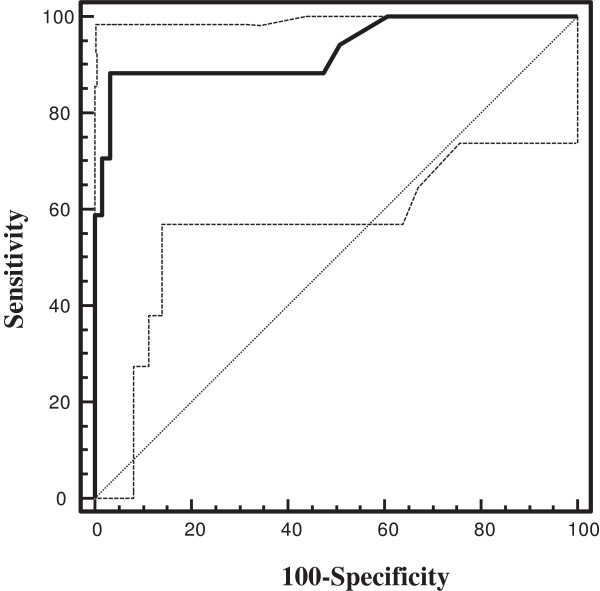
**ROC analysis of anti-type VII collagen IgG.** Receiver-operating-characteristic (ROC) plot analysis of anti-type VII collagen IgG autoantibodies. AUC, area under the curve, was 0.931. The continuous line refers to the ELISA method used.

The area under the curve (AUC) for anti-type VII collagen ELISA test was 0.931(CI, 0.850-0.976) and ROC analysis showed that anti-type VII collagen autoantibodies had a sensitivity of 88.2% (CI, 63.5 -98.2) and a specificity of 96.7% (CI, 88.6-99.5), using a cut-off value of 5 UA/mL (Figure 2). The prevalence of anti-type VII collagen autoantibodies was 88% in patients with RDEB and 10% in patients with EBS. The combined prevalence of anti-BP180 and anti-BP230 autoantibodies was 88% in patients with RDEB and 50% in patients with EBS.

Thus, in RDEB forms there was a higher prevalence of both anti-type VII collagen autoantibodies and autoantibodies against BMZ antigens, in particular BP180 and BP230 (chi-square, P < 0.0001; OR 79.95% IC 4.01-1575).

### Analytical and clinical correlations

ELISA results for each autoantibody and BEBS score for each patient are reported in Table 2.

**Table 2:** ELISA data for each autoantibody and BEBS score for each patient with recessive dystrophic epidermolysis bullosa (RDEB) and with epidermolysis bullosa simplex (EBS)

				**Autoantibodies anti-skin**			
**n. pt**	**Sex**	**Age years**	**tipe EB**	**Anti-BP180**	**Anti-BP230**	**Anti-coll VII**	**BEBS score**
1	M	20	RDEB	68,8	45,2	27,9	65
2	M	12	RDEB	36	26,7	20,1	67
3	F	4	RDEB	23,5	20,3	8,5	56
4	F	6	EBS	14,7	13,8	2,1	2
5	F	19	RDEB	37,5	37,8	14,9	63
6	F	45	RDEB	6,8	3,3	1,1	39
7	M	34	RDEB	93	53,4	24,5	79
8	M	34	EBS	2,9	3,2	1,3	2
9	F	10	EBS	3,5	3,5	1,2	2
10	F	27	RDEB	1,4	110	53,5	89
11	F	15	RDEB	77,8	69,8	44,4	60
12	F	29	RDEB	151,8	144,5	48,6	82
13	F	29	RDEB	34,5	18,2	5,9	53
14	M	9	RDEB	16,8	13,9	5,8	55
15	F	7	RDEB	76,7	57,9	29,9	53
16	F	7	EBS	13,4	3,5	1,4	2
17	F	7	EBS	10,1	6,3	1,9	2
18	F	33	RDEB	107,6	65,9	45,3	90
19	M	19	EBS	2,7	4,1	1,2	2
20	F	56	EBS	15,1	13,1	10,8	36
21	F	50	RDEB	2,8	3,3	1,2	11
22	F	16	RDEB	161	120,7	76,7	71
23	F	10	RDEB	58,2	52,8	36,6	52
24	F	17	EBS	4,5	8,9	2,1	2
25	M	13	EBS	15,1	8,4	2,2	2
26	F	2	RDEB	56	27,1	12,4	34
27	F	1	EBS	1	1,2	1,4	2

**Figure 3:**
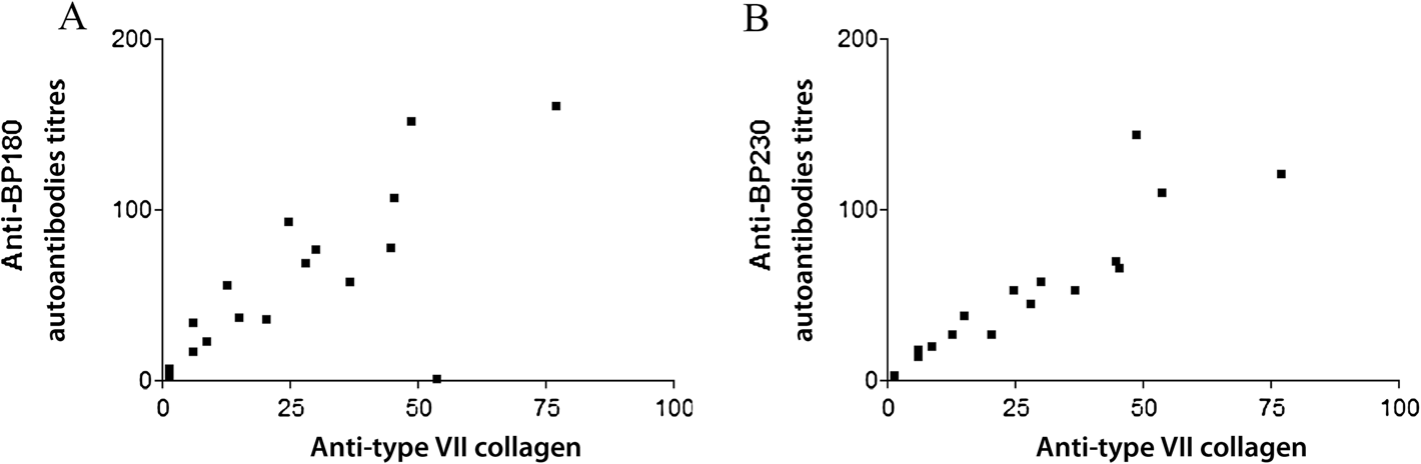
**Correlation between anti-type VII collagen autoantibodies titres and the other two anti-skin autoantibodies. (A)** Correlation between anti-type VII collagen autoantibodies titres and anti-BP180 autoantibodies. Pearson test, P < 0.0009, R^2^ 0.53. **(B)** Correlation between anti-type VII collagen autoantibodies titres and anti-BP230 autoantibodies. Pearson test, P < 0.0001, R^2^ 0.83.

In all patients with RDEB, anti-type VII collagen autoantibodies titres significantly correlated with both anti-BP180 (slope < 0.0009, R^2^ 0.53) (Figure 3A) and anti-BP230 (slope < 0.0001, R^2^ 0.83) autoantibodies titres (Figure 3B).

**Figure 4:**
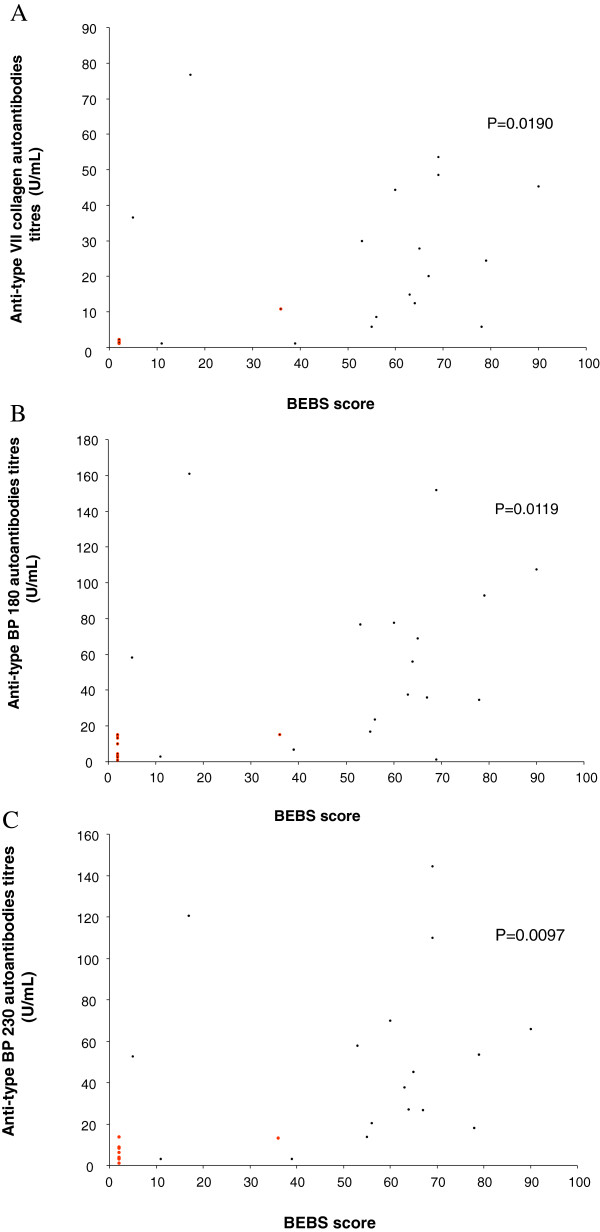
**Correlation between anti-skin autoantibodies and Birmingham Epidermolysis Bullosa Severity score. (A)** Correlation between anti-type VII collagen autoantibodies titres and BEBS score. **(B)** Correlation between anti- anti-BP180 autoantibodies titres and BEBS score. **(C)** Correlation between anti-BP230 autoantibodies titres and BEBS score. Red points indicate EBS patients; black points indicate RDEB patients.

Birmingham Epidermolysis Bullosa Severity score (BEBS) correlated with anti-type VII collagen autoantibodies titres (P = 0.0190) (Figure 4A) and also with anti-BP180 and anti-BP230, P = 0.0119 and P = 0.0097, respectively (Figure 4B and 4C).

## Discussion

Over the last years, the great worldwide interest in inherited EB has led to the publication of studies concerning EB clinical features and its genetic correlation, which are important for the disease classification and the prediction of organ involvement. However, no studies about serological features of patients with different subtypes of inherited EB are available. Recently, recombinant proteins have been used as antigenic targets in ELISA methods for detection of anti-skin autoantibodies. These methods have also confirmed that the sensitivity and the specificity are analogous to those of the method of reference.

In our study, we analysed for the first time in the literature the presence of circulating anti-skin autoantibodies in patients suffering from RDEB and EBS. In other autoimmune blistering skin diseases (as pemphigus and pemphigoid), it has been shown that anti-skin specific autoantibodies can take part in the formation process of the bullous lesion [19]. Regarding EBA, the cross-reacting autoantibodies are responsible for the incorrect assembly of type VII collagen into anchoring fibrils, thus altering both the natural maintenance and turnover of fibrils or interfering with the ability of the anchoring fibrils to attach to the basement membrane at dermal level [29].

The pathogenic role of these autoantibodies has been demonstrated in experimental models [30].

As for DEB, relatively little is known about the mechanistic consequences of the mutations in relation to blistering, which has led us to look for autoantibodies directed against cutaneous structures also in these patients. Some very interesting data have emerged from our study about the prevalence of anti-skin positivity in patients with RDEB.

The relationship between a monogenic disease and autoimmunity is difficult to clarify; we tried to explain it through different hypothesis.

In RDEB patients, a possible explanation for the development of autoantibodies recognizing type VII collagen epitopes could be based on an altered immunologic recognition of "self" consequence of altered proteins synthesis due to genetic mutation.

We know that autoimmune diseases are characterized by aberrant immune responses against healthy cells and tissues, in which a given individual's genetic susceptibility may play a central role. However, the exact mechanisms underlying the development of these conditions remain for the most part unknown. In recent years, growing evidence has demonstrated that, in addition to genetics, other complementary mechanisms are involved in the pathogenesis of autoimmunity; in particular, epigenetics. Epigenetics is defined as stable and heritable patterns of gene expression that do not entail any alterations to the original DNA sequence. Epigenetic mechanisms primarily consist of DNA methylation, histone modifications and small non-coding RNA transcripts [31].

Another hypothesis for this autoimmune condition may relate to the "molecular mimicry", based on the assumption that a foreign antigen holds similar sequence or structure with self-antigens. Molecular mimicry has typically been characterized at antibody or T cell level. Moreover, the exposure to antigen protein derived from bacterial and viral infection can induce this phenomenon with the activation of T and B cells [32]. RDEB patients, as a consequence of chronic wound formation, are often exposed to bacterial infection; therefore, molecular mimicry is likely to occur. However, it is possible that the initial insult to the anchoring fibrils, mediated by an antibody recognizing a single epitope, could lead to the exposure of additional epitopes and subsequent development of polyvalent antisera.

However, it has recently been shown that the reactivity versus the NC-1 and NC-2 domains of type VII collagen protein is more predictive of an inflammatory rather than an autoimmune disease. This hypothesis is supported by the presence of anti-type VII collagen autoantibodies in inflammatory diseases involving the intestine, where the presence of this protein has been demonstrated but not featured by skin blistering such as Crohn’s disease and inflammatory bowel disease. However, the correlation between the presence of those antibodies and their pathogenic significance in that illness remains elusive [33,34]. It has recently been reported that the major determinant of autoantibody pathogenicity relies on the different IgG subclass, and within the anti-type VII collagen autoantibodies the IgG4 sub-class was the most represented. IgG4 may induce inflammation and tissue damage by activating leukocytes with a non-complement fixing pattern or just binding collagen VII in a Fc-independent fashion leading to dermal-epidermal separation [35]. Although in our study we have not identified the subclass of IgG-autoantibodies, we observed very high levels of IgG4 among patients with RDEB; this observation might explain the dissimilar clinical evolution not only relative to skin manifestation, but also in relation to extracutaneous complication, such as organ damage.

In this study, we have also found autoantibodies against NC-16A domain of BP180 and C- and N-terminal domains of BP230. Another current model of chronic autoimmune skin diseases predicts that “dominant” self epitopes induce the inflammatory cascade and tissue damage in the target organ. Recruitment and activation of autoreactive T lymphocytes specific for “secondary” epitopes of the nominal autoantigen or non distinct self-proteins subsequently contribute to the disease course. This phenomenon is called epitope spreading (ES). There is growing evidence that inter- and intramolecular ES events are not simply an epiphenomenon but are critical in the evolution and progression of diseases [36]. Our data confirm this hypothesis and demonstrate that anti-type VII collagen autoantibodies strictly correlate with BP180 and BP230 autoantibodies.

Our study could be limited by the lack of Direct Immunofluorescence (DIF) data on skin biopsies for detection of tissue-bound autoantibodies. We believe that this detection, which is very important to define the role of these autoantibodies, should be carried out by more specific epitope-related methods rather than by fluorescence pattern observed with DIF.

Our opinion is that the most significant finding can be seen in the correlation between antibodies titres and the skin disease activity score. However, we also think that this finding should be confirmed on a wider number of DEB patients, considering the natural history of the disease. These correlations can also be assessed by means of other clinical scores [37-39]. On the other hand, the determination of anti-skin autoantibodies by means of the ELISA method has many advantages: it can be easily introduced in the clinical routine, it is little traumatic for the patient, therefore repeatable, and quantitative.

## Conclusions

In this study, we have reported the presence of skin autoantibodies in EB patients. In particular, we revealed a high prevalence of autoantibodies in RDEB patients. Unless the pathogenic role of antiskin autoantibodies in patients with DEB is demonstrated by ex vivo and experimental animals using antibody passive transfer model, their presence can be simply interpreted as an epiphenomenon.

Experimental models can be very useful not only to investigate the mechanism underlying pathogenetic autoantibody production and autoantibodies mediated tissue injury, but also for the development of more effective therapeutic strategies for this disease.

## Abbreviations

EB: Epidermolysis bullosa; RDEB: Recessive dystrophic epidermolysis bullosa; EBS: Epidermolysis bullosa simplex; IIF: Indirect immunofluorescence; ELISA: Enzyme linked immunosorbant assay; BP: Bullous pemphigoid; JEB: Junctional epidermolysis bullosa; DEB: Dystrophic epidermolysis bullosa; BMZ: Basement membrane zone; IFM: Immunofluorescence mapping; TEM: Transmission electron microscopy; NC: Non-collagenous; EBA: Epidermolysis bullosa acquisita; IgG: Immunoglobulin G; MMP: Mucous membrane pemphigoid; BEBS: Birmingham epidermolysis bullosa severity score; PV: Pemphigus vulgaris; ICS: Intercellular substance; RT: Room temperature; OD: Optical density; ROC: Receiver operating characteristics; AUC: Area under the curve; CI: Confidence interval; OR: Odds ratio; ES: Epitope spreading.

## Competing interests

The authors declare that they have no financial and no-financial competing interests.

## Authors’ contributions

MT and GA conceived and designed the study; MT performed the assays and carried out the statistical analysis; LG, GA and AF obtained the clinical data; GA, DB and LB interpreted the data; MT, AF and MGM wrote the paper; CDG made the linguistic revision. All authors read and approved the final manuscript.
